# Using *RELION* software within the *Scipion* framework

**DOI:** 10.1107/S2059798321001856

**Published:** 2021-03-30

**Authors:** Grigory Sharov, Dustin R. Morado, Marta Carroni, José Miguel de la Rosa-Trevín

**Affiliations:** a MRC Laboratory of Molecular Biology, Cambridge Biomedical Campus, Cambridge, United Kingdom; b Department of Biochemistry and Biophysics, Science for Life Laboratory, Stockholm University, Stockholm, Sweden

**Keywords:** *Scipion*, *RELION*, image processing, cryo-EM, single-particle analysis

## Abstract

*Scipion* is a modular image-processing framework that integrates several software packages under a unified interface while taking care of file formats and conversions. Here, new developments and capabilities of the *Scipion* plugin for the widely used *RELION* software package are presented and illustrated with an image-processing pipeline for published data. The user interfaces of *Scipion* and *RELION* are compared and the key differences are highlighted, allowing this manuscript to be used as a guide for both new and experienced users of this software. Different on-the-fly image-processing options are also discussed, demonstrating the flexibility of the *Scipion* framework.

## Abstract 2

An overview of the *Scipion* plugin for the *RELION* software package is presented and various image-processing capabilities within the *Scipion* framework are discussed.

## 1. Introduction   

The cryogenic electron microscopy (cryo-EM) field continues to grow rapidly due to constant improvements in both instrumentation and software algorithms (Danev *et al.*, 2019 ▸). With an increasing number of high-resolution structures being solved every year (https://www.ebi.ac.uk/pdbe/emdb/statistics_main.html/) by single-particle analysis (SPA) and sub­tomogram averaging methods, the automation of existing image-processing pipelines becomes more relevant (Biyani *et al.*, 2017 ▸; Burnley *et al.*, 2017 ▸; Tegunov & Cramer, 2019 ▸; Stabrin *et al.*, 2020 ▸). Moreover, the introduction of user-friendly graphical interfaces (GUIs) helps to guide new or less experienced users through the complete process of high-resolution cryo-EM and makes the software more accessible. *Scipion* (de la Rosa-Trevín *et al.*, 2016 ▸) is an integrative software suite that aims to reach a larger scientific audience by providing a convenient modular platform for building custom automatable pipelines. The platform can be adapted to suit the specific image-processing needs of structural biologists, electron-microscopy facilities or software developers. The *Scipion* framework includes multiple modules (plugins), each providing a set of Python wrappers around particular cryo-EM software. *Scipion* collects these modules within a unified GUI, allowing a seamless transition between different packages and file formats. Here, we describe in detail the *Scipion* plugin for the *RELION* software package (Zivanov *et al.*, 2020 ▸) and illustrate the advantages of image processing using the *Scipion* platform.

## 2. Plugin overview   

With the high popularity of *RELION* within the cryo-EM community (https://www.ebi.ac.uk/pdbe/emdb/statistics_main.html/), the plugin for the *RELION* software package is one of the most widely used by *Scipion* users (http://scipion.i2pc.es/report_protocols/protocolTable/). Recently, the plugin has undergone several important changes. Firstly, the code has been migrated to Python 3, since Python 2.7 was deprecated in January 2020. While being entirely transparent to users, this transition has brought many improvements for software developers and allowed us to keep the code base up to date. Secondly, a new STAR-file parser *emtable* (de la Rosa-Trevín & Sharov, 2020 ▸) has been developed to simplify and speed up metadata conversion between *Scipion* and *RELION*, replac­ing the functions of the plugin for *XMIPP* (de la Rosa-Trevín *et al.*, 2013 ▸) that was previously used for this task. *Emtable* is available as a small self-contained Python module and can be used to manipulate STAR files independently from *Scipion*.

Considerable effort has been put into compatibility between different *RELION* versions. Starting with *RELION* version 3.1, information about optics groups has been added as a second data table to STAR files. Optics groups were designed to keep all information related to data acquisition (pixel size, voltage *etc.*) separate from the particle metadata and to allow users to combine different data sets. Despite the fact that we no longer provide support for *RELION* 3.0 or older versions, users are still able to import older STAR files into *Scipion* and then continue with *RELION* 3.1. We have created a special protocol called ‘assign optics groups’ to account for the differences between the *Scipion* EM data model and the new optics groups data table. Users can assign the parameters of one or more (using an extra STAR file) optics groups to a set of images at essentially any step of the image-processing pipeline. This allows more flexibility compared with the *RELION* GUI, where users have to specify optics groups at the import step.

At the moment, the plugin provides wrappers for most of the programs available from *RELION* (version 3.1), with the exception of the protocols for helical image processing (He & Scheres, 2017 ▸) and subtomogram averaging (Bharat *et al.*, 2015 ▸), which are still in development.

## 3. User-interface design   

**Figure 1:**
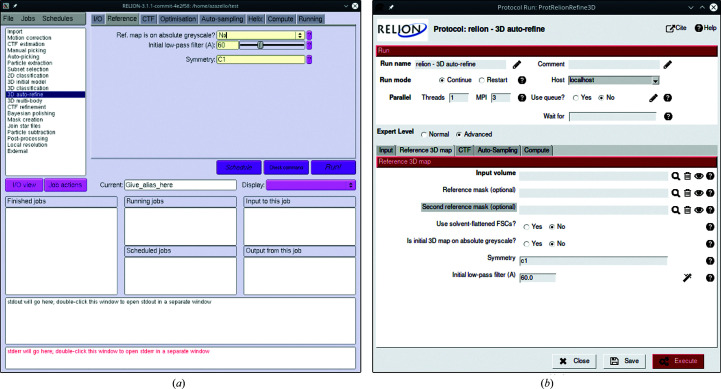
Comparison of the 3D auto-refine protocol GUIs in *RELION* (*a*) and *Scipion* (*b*). Advanced protocol parameters in *Scipion* are highlighted with a gray background.

The original idea behind a *Scipion* wrapper for *RELION* programs was to make the user interface similar to the original *RELION* GUI so that *RELION* users starting to use *Scipion* could easily orient themselves through many available options. Below, we describe the interface of the plugin for the *RELION* software package and compare it with the *RELION* GUI (Fig. 1 ▸).

Every protocol window in *Scipion* has a similar layout composed of two parts. The top panel includes computation-related parameters such as submission to a cluster queue, a waiting list (the current protocol will not start until the listed protocols have finished) and parallelization options (GPU IDs, number of MPI processes and/or threads) if the protocol supports them. Furthermore, many protocols define two levels of user expertise: normal and advanced (see ‘Expert Level’ in Fig. 1 ▸). Selecting the advanced level displays additional parameters that are less common or reserved for special cases. Many plugin protocols also offer a string-type variable called ‘Extra parameters’ or ‘Additional arguments’. Such strings are reserved for *RELION* command-line options and arguments that are not available through the standard GUI and can be utilized by advanced *RELION* users.

**Figure 2:**
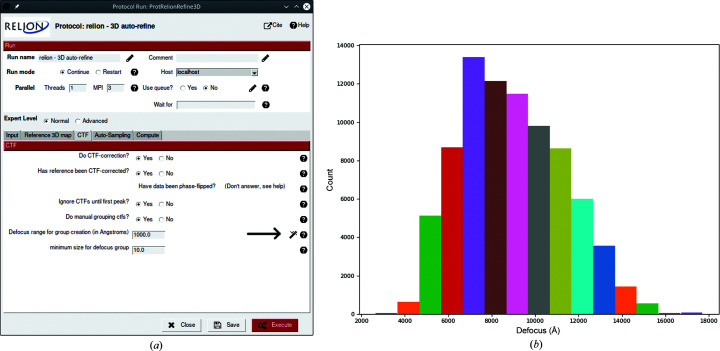
The plugin for the *RELION* software package provides interactive wizards to help users choose optimal parameters. The arrow in (*a*) indicates the wizard button that launches a histogram plot (*b*) with an estimated number of CTF groups given the input defocus range.

The bottom panel of the protocol window includes task-specific parameters as found in the *RELION* GUI, organized in similarly named tabs. Some of the parameters have an interactive wizard attached (Fig. 2 ▸), meaning that one can quickly visualize how changing certain values might affect the results. Most wizards are used to select a particle mask size or to choose image-filtering levels; however, there are also special cases such as choosing a detector MTF file or creating defocus groups.

Despite many similarities with the *RELION* GUI, the plugin has several differences due to the conceptually different design of the *Scipion* framework. One example is the ‘continue’ mode for classification/refinement protocols. While *RELION* allows the continuation of a finished job, in *Scipion* every run is unique and the user must create a new protocol which should reference the previous one. In this way, the meaning of the number of iterations becomes different between *RELION* and *Scipion*: for *RELION* it is a total number of iterations, but for *Scipion* it is a number of extra ‘continue’ iterations.

**Figure 3:**
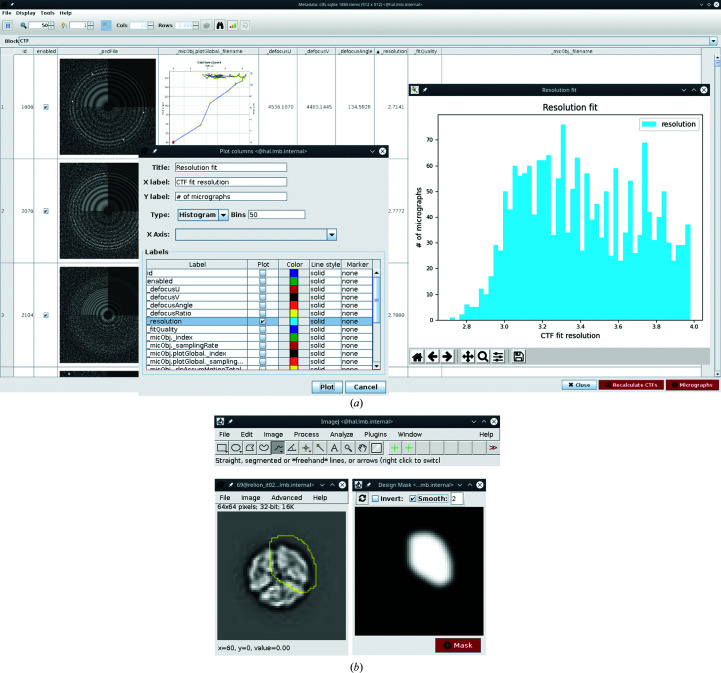
*Scipion* offers a rich viewing interface for both item sets and individual images. (*a*) *RELION* STAR-file metadata can be sorted and plotted in various ways. (*b*) Individual images can be analyzed and processed with built-in *ImageJ* tools. Here, an interactive wizard for 2D mask design is depicted.

Another example is subset selection, which is performed in *RELION* with a separate job type. In *Scipion* subsets can be created naturally from any set of items in an interactive manner. *Scipion* uses the *XMIPP* viewer to display sets of images and associated metadata (Fig. 3 ▸ *a*). These sets can be displayed as an image gallery or as a table, in which the values in each column can be sorted or plotted using built-in tools. This means that any STAR file can be visualized with *Scipion* without using a text editor. Moreover, *Scipion* includes a set of protocols that can perform various operations on image sets such as joining, splitting or intersection. When displaying single items such as a micrograph or a particle, *Scipion* offers a range of *ImageJ* (Schneider *et al.*, 2012 ▸) image-processing tools that can be used, for example, to calculate a fast Fourier transform (FFT) or to interactively create a 2D mask from an image (Fig. 3 ▸ *b*).

The plugin for the *RELION* software package also includes custom-designed viewers that not only let users visualize output sets of items from a given protocol but also create various plots for FSC, SSNR, *B* factors, angular distribution and other metrics to quickly follow job progress and iteration results. Protocols for refinement and classification provide the possibility to track changes between iterations, such as orientations, offsets or number of images per class. Other examples include frame-motion plots for movie-alignment protocols and defocus-variation plots for CTF refinement protocols. Combined with the possibilities of creating custom plots from STAR file-derived metadata and displaying volumes in *ChimeraX* (Pettersen *et al.*, 2021 ▸), the plugin for the *RELION* software package offers a rich interactive interface for both novice and expert users.

## 4. On-the-fly processing   

On-the-fly processing (or streaming) is often used for more efficient data collection by providing rapid feedback on data quality. It can also considerably shorten the data-processing time by overlapping it with microscope acquisition. *Scipion* provides various options for running workflows in streaming mode, some of which are discussed below.

Firstly, most import protocols have a ‘Streaming’ tab where users can specify a file timeout and a global timeout. The former is used to recognize when the input file size stops growing and is ready to be imported, while the latter is used to stop streaming once no new data are being acquired. Secondly, for several plugins that can run on GPUs *Scipion* provides the possibility of parallel multi-GPU execution. Such examples are *MotionCor*2 (Zheng *et al.*, 2017 ▸) for movie alignment and *Gctf* (Zhang, 2016 ▸) for CTF estimation. By specifying a number of threads equal to the number of GPUs plus one, a user can submit streaming jobs to several GPUs simultaneously. For instance, setting the number of threads to 3 and the GPU IDs to ‘0, 1’ will lead to thread 1 being a master, while thread 2 will run on GPU 0 and thread 3 on GPU 1. Each worker thread will process a single movie in streaming, giving a speed boost in processing. More complex combinations are also possible but depend on the underlying protocol.

Multi-GPU execution, however, is different for *RELION* protocols, where a user usually has to specify both MPI processes and threads. We have left the syntax for GPU IDs the same as in the original *RELION* GUI to avoid confusion. Users can refer to the *RELION* website documentation for more details.

The protocols for CTF estimation, particle extraction and auto-picking in most *Scipion* plugins also have a ‘Streaming’ tab where a user can provide a batch size. By default (batch size 1), all input protocol items will be processed one by one, which is common for on-the-fly processing pipelines. However, users can specify a larger number of items that will be then processed ‘in batches’, which can sometimes decrease the computational load and speed up processing. Setting this value to 0 is used for nonstreaming cases when all input items are processed together.

Altogether, multiple plugins in *Scipion* support various streaming scenarios for most image-preprocessing steps up to 2D classification. Such protocols can be found in the protocol search window of the *Scipion* project (available by pressing Ctrl+F). Below, we show an example of streaming processing on real data.

## 5. Interaction with other software   

Besides the ability to import data from various cryo-EM packages provided by *Scipion* itself, the plugin for the *RELION* software package includes several protocols that can export results into a self-contained folder, allowing users to continue image processing outside *Scipion*. Export at the level of coordinates, CTFs (micrographs with CTF information) and particles is possible. We are working towards running all export protocols in streaming so that the results can be immediately accessed by other software packages or by *RELION* itself (outside *Scipion*).

From the beginning, the *Scipion* framework has always been focused on providing a transparent combination of different software packages. As a result, *RELION* users are free to incorporate various programs for movie alignment, CTF estimation and particle picking into a standard *RELION* pipeline. Global movie-alignment parameters produced by *MotionCor*2 (Zheng *et al.*, 2017 ▸) or *Unblur* in *cisTEM* (Grant *et al.*, 2018 ▸) can be used as input for Bayesian polishing in *RELION*. Moreover, the plugin includes several protocols which do not have their own GUI in the *RELION* interface. Such examples are the centering of class averages, movie compression, gain estimation for movie compression, volume symmetrization and several others.

## 6. Image processing   

**Figure 4:**
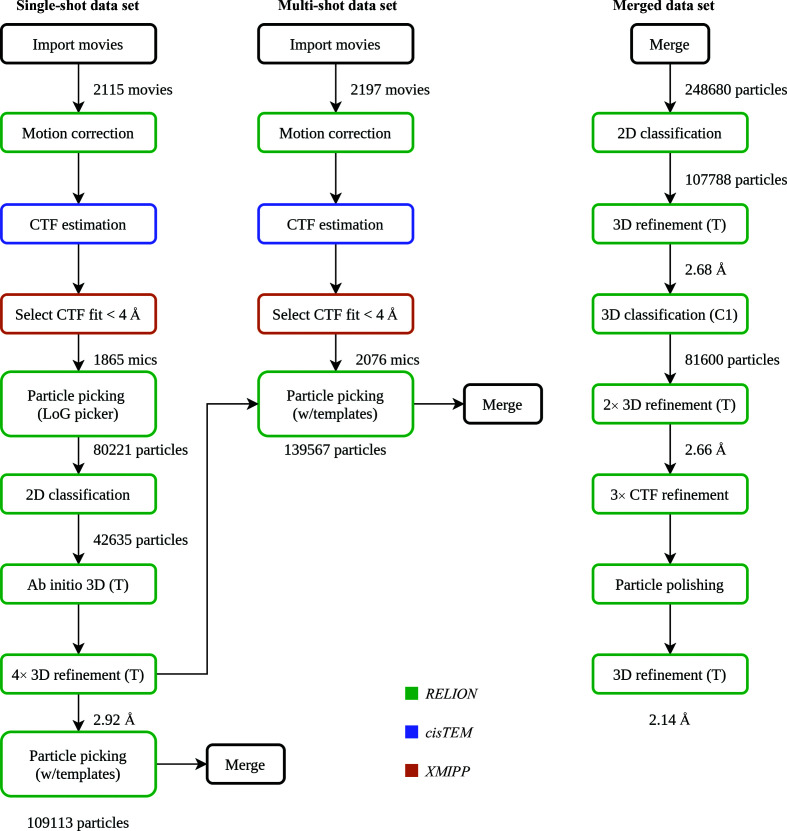
Simplified flow chart of *RELION* processing within the *Scipion* framework for the EMPIAR-10389 data set. We first processed the single-shot data set separately and then used the resulting map for the reference-based particle picking of both single-shot and multi-shot data sets (see the text for details). At this point the two data sets were joined and processed further together. For comparison, see Supplementary Fig. S2 in Righetto *et al.* (2020 ▸) and the supporting information to this manuscript.

To demonstrate the capabilities of the plugin for the *RELION* software package, we have chosen the EMPIAR-10389 data set (Righetto *et al.*, 2020 ▸). The deposited data set consists of two sets of movies: one acquired with beam-image shift (multi-shot) and one without (single-shot). The published workflow leads to a 2 Å resolution map and comprises a wide range of protocols including the basic steps from preprocessing to 3D refinement, along with more complex image-processing tasks such as changing the data-set binning, particle re-centering, joining of different data sets, particle polishing and CTF refinement. We think that this workflow provides a good example to illustrate the capabilities of *Scipion* for image processing. Even though based predominantly on *RELION*, this example shows the advantages of performing these operations within the *Scipion* framework rather than using the *RELION* GUI. To reduce the computational costs, we have simplified the pipeline by removing several intermediate steps; however, the general workflow outline remains the same (Fig. 4 ▸). To validate the reproducibility of results between *RELION* and *Scipion*, we first ran this workflow in the *RELION* 3.1 GUI (outside *Scipion*) to replicate the steps that the authors used in their publication to the best of our knowledge. Both procedures are described below.

### 6.1. Processing in *RELION* outside *Scipion*   

The raw movies were downloaded from EMPIAR (Iudin *et al.*, 2016 ▸), split into four optics groups based on the movie filename (three multi-shot groups and one single-shot group) and imported separately into the *RELION* project. Movie STAR files for multi-shot groups were joined together, and then both multi-shot and single-shot data sets were processed in the following manner. Movies were motion-corrected with *RELION* *MotionCorr* (no frame grouping, using 5 × 5 tiles) and amplitude power spectra sums were produced for further CTF estimation with *CTFFIND* 4.1.14 (Rohou & Grigorieff, 2015 ▸). Micrographs with an estimated CTF fit lower than 4 Å were excluded from further processing. The single-shot data set was first processed separately.

Initially, the *LoG-picker* algorithm was used to pick particles, which were then extracted using a 512-pixel box size and binned eight times to a pixel size of 5.11 Å in order to speed up the downstream processing. Around 80 000 binned particles were classified into 80 2D classes with the ‘Ignore CTF until first peak’ option enabled. Particles from the best 2D classes showing relevant high-resolution features were regrouped (using the ‘Subset selection’ job) and subjected to the stochastic gradient-descent algorithm implemented in *RELION* to generate an *ab initio* 3D map (applying tetrahedral symmetry). The resulting map was used as a reference for masked 3D refinement that reached the Nyquist limit for the binned data. The particles were then re-extracted with binning four times using re-centered coordinates by applying shifts from the refinement, and the 3D refinement was repeated. Re-extraction and refinement cycles were repeated until the unbinned box size was used, leading to a 2.84 Å resolution reconstruction from ∼45 000 particles. In comparison with the publication, we did not perform further refinements, 3D classification or Bayesian polishing for this data set.

Subsequently, we used a reference-based *RELION* particle picker with the 3D reference map from the last 3D refinement with the data binned twice. We picked approximately 97 000 and 129 000 particles from the single-shot and multi-shot data sets, respectively. The particles were separately extracted, joined and 2D classified (again binned eight times with a 64-pixel box size to speed up calculations). The particles were then extracted again (binned twice) and compared with their counterparts (binned eight times). Here, we had to modify the particle STAR file manually so that it would point to the best particle subset after the 2D classification but with a different binning level. This particle subset was submitted to a 3D refinement that led to a 2.66 Å resolution structure from a total of ∼110 000 particles. Next, we performed a masked 3D classification (without imposing symmetry) without alignment to further clean the data. Finally, all particles from the best-looking and largest 3D class were re-extracted unbinned and refined to a 2.64 Å resolution map. We continued the processing with three sequential rounds of CTF refinement to correct for magnification anisotropy, to refine the defocus per particle and astigmatism per micrograph, and to correct for high-order aberrations, respectively. This was followed by Bayesian particle polishing using the parameters from the published manuscript. A final round of 3D refinement and post-processing led to a 2.11 Å resolution map.

### 6.2. Processing in *Scipion*   

After completing the workflow in the *RELION* GUI, we switched to the *Scipion* plugin for the *RELION* software package. Here, all protocol parameters were set to be consistent with the *RELION* values used above. Similarly, we first focused on the single-shot data set. Gain references and defect files were downloaded from EMPIAR in advance and the preprocessing was performed in streaming mode. As the movies were being downloaded, the following steps were executed for each movie: import, *RELION* motion correction, CTF estimation with *CTFFIND*4 (via the plugin for *cisTEM*), sorting micrographs by CTF fit (via the ‘ctf consensus’ protocol of the plugin for *XMIPP*), *RELION* LoG picking and particle extraction.

*Scipion* offers several options for the execution of the workflow: (i) importing a predefined workflow file in JSON format into a *Scipion* project and then running it (performed via the *Scipion* GUI or through the command line; https://scipion-em.github.io/docs/docs/facilities/facilities-workflows.html), (ii) scheduling protocols to run one after another using the waiting-list option or (iii) creating a workflow interactively: as soon as a single output item of one protocol appears, it can be connected to another protocol as input. Additionally, it is also possible to process items in batches in streaming mode as described earlier. Here, we used a combined approach and set up *Scipion* to run import and motion correction on a one-by-one item basis, while CTF estimation, picking and extraction were run in batches on every 20 micrographs. Further processing of the single-shot data set up to the final 3D refinement with unbinned data was performed using the exact same job parameters as for *RELION*. Rescaling of 3D reference volumes was performed with the plugin for *XMIPP*. Particle re-extraction was performed in a different way compared with *RELION*. Currently, the particle-extraction protocol in the plugin for the *RELION* software package does not support input particles, so a user must run an ‘extract coordinates’ protocol, which produces a new set of coordinates taking into account particle shifts, in our case from the 3D refinement. This is followed by a normal particle-extraction step. Final 3D refinement for the single-shot data set reached 2.87 Å resolution.

**Figure 5:**
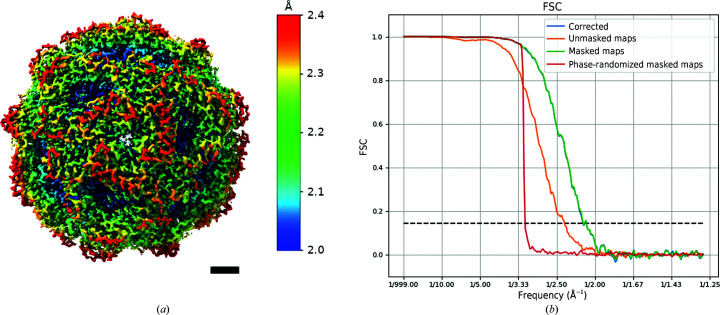
Final results produced by the *Scipion* plugin for the *RELION* software package. (*a*) The cryo-EM map filtered and colored by local resolution. The scale bar is 20 Å in length. (*b*) Fourier shell correlation curves between the half-maps.

For the multi-shot data set the preprocessing was performed in a standard non-streaming way as joining item sets is currently not possible on the fly in *Scipion*. Similarly to the *RELION* workflow described above, particles from both single-shot and multi-shot data sets were picked using a 3D reference volume from the previous 3D refinement with the data binned twice. The eight times binned particles from both data sets were extracted, joined and submitted to 2D classification. The twice-binned particles were then re-extracted, joined and cross-referenced with their more binned counterparts using the *pwem* subset protocol with intersection option. Here, no STAR-file editing was required to compare particles with different binnings or to select the best subset after classification. *Scipion* is able to compare any two item sets as long as they belong to the same ‘type’, in our case, particles. Afterwards, 3D classification without alignment, followed by the re-extraction of unbinned particles from the best class and subsequent 3D refinement, was performed, resulting in a 2.62 Å resolution structure. Before starting CTF refinements we used the *RELION* assign optics protocol, providing a simple STAR file containing two columns, movie name and optics group number as input to tell *RELION* that we have four different beam-tilt classes in our combined particle set. When using the *RELION* GUI this step is performed at the import stage or by manually editing the STAR files. In *Scipion* we provide a semi-automated solution in which optics groups can be assigned at any step of the workflow. Once all three CTF refinement rounds had been completed, we joined the single-shot and multi-shot movies after motion correction and assigned optics groups again (now to the movies), since particle polishing in *RELION* also requires movies as input. The final 3D refinement with polished particles reached 2.14 Å resolution (Fig. 5 ▸). We then compared the maps produced by *RELION* workflows inside or outside *Scipion*. After importing both maps into *Scipion* and aligning them to each other (the hand of the *Scipion* map had to be flipped) using the ‘align volume’ protocol of the *XMIPP* plugin, we calculated the difference map (‘operate volumes’ protocol), which showed no significant features. Moreover, on visual inspection we find that the resolution difference between the two maps is negligible, which is expected as the *Scipion* wrapper essentially executes the exact same *RELION* commands provided that input parameters are the same. Minor variations can be attributed to differences in user-based class or particle selections, different 3D refinement starting seeds or small variations in the application of shifts during particle re-extraction. We have also compared both of our maps with EMDB entry EMD-10835 deposited by the original authors. The high-resolution features of their 1.98 Å resolution map are more pronounced than in our 2.14 Å resolution map (Supplementary Fig. S1). We believe this difference to be caused by the fact that in our simplified workflow we did not iterate 3D refinements after each CTF refinement step, which could have improved the angular accuracy and resolution.

## 7. Conclusions   

Here, we describe the functionality of the *Scipion* plugin for the *RELION* software package, with a focus on single-particle image processing for cryo-EM. The plugin provides a set of Python wrappers for the *RELION* programs, while at the same time offering all of the capabilities of *Scipion*’s image and metadata handling via a user-friendly graphical interface. We compare the *RELION* and *Scipion* interfaces and demonstrate using published raw data how the *Scipion* plugin for the *RELION* software package can be used to execute a complete SPA image-processing workflow. Further documentation on the *Scipion* software can be found at https://scipion-em.github.io/docs/. The plugin code is freely available on Github (https://github.com/scipion-em/scipion-em-relion) and is distributed under the GNU general public license (GPL3). For user questions and feedback, please use Github Issues or the *Scipion* mailing list at https://sourceforge.net/projects/scipion/lists/scipion-users.

## 8. Data availability   

The map produced by the *Scipion* workflow has been deposited in the EMDB with accession code EMD-12236. The associated workflow has been uploaded to http://workflows.scipion.i2pc.es/.

## Supplementary Material

EMDB reference: reprocessed EMPIAR-10389 data (urease), EMD-12236

Supplementary Figure S1 and differences from the published EMPIAR-10389 workflow. DOI: 10.1107/S2059798321001856/ic5115sup1.pdf
