# Supplementary material for: Using RELION software within the Scipion framework
Source: Acta Crystallogr D Struct Biol. 2021 Mar 30;77(Pt 4):403–10. doi: 10.1107/S2059798321001856 (PMC8025880; doi:10.1107/S2059798321001856)
Supplement: Supplementary file 1 [file d-77-00403-sup1.pdf]

STRUCTURAL
BIOLOGY

Volume 77 (2021)

Supporting information for article:

Using *RELION* software within the *Scipion* framework

Grigory Sharov, Dustin R. Morado, Marta Carroni and José Miguel de la Rosa-Trevín

Differences from the published EMPIAR-10389 workflow

1. Single-shot processing was simplified: we did not perform further refinements (CTF, polishing, classification, etc.) after the first 3D refinement.

2. We have used Relion reference-based picker with the 3D volume as a reference, while in the original manuscript Gautomatch (Kai Zhang, https://www2.mrc-lmb.cam.ac.uk/research/locally-developed-software/zhang-software/) was used.

3. We have used the Relion implementation of motioncorr instead of motioncor2. 5x5 patches and no frame grouping were used in our case. Additionally, amplitude power spectra were calculated and used for CTF estimation, while in the manuscript authors used aligned movies for CTF estimation.

4. The deposited EMPIAR dataset does not contain movies for which the CTF resolution fit was worse than 4Å.

5. We used solvent-flattened FSC during all 3D refinements.

6. For the merged dataset we did not perform 3D refinements after each CTF refinement step.

7. During CTF refinement we have opted to refine astigmatism only per micrograph and not per particle.

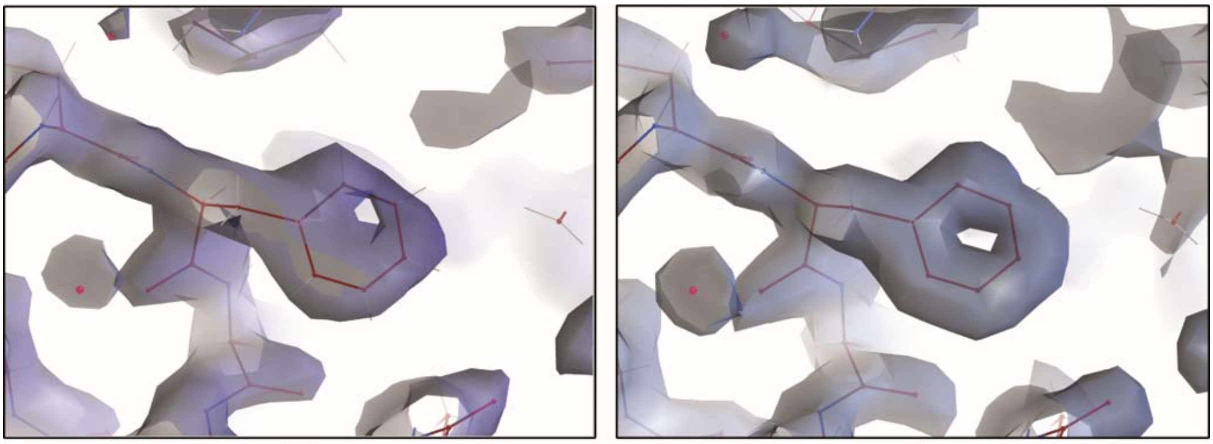

(*a*) (*b*)

Figure S1 Comparison of high-resolution features (around B74, PHE) between our 2.14Å map (a) and 1.98Å EMDB-10835 map (b). Both maps show densities for water molecules, however the hole in the aromatic ring of the PHE residue looks more pronounced.
